# Answers to Health Questions: Internet Search Results Versus Online Health Community Responses

**DOI:** 10.2196/jmir.5369

**Published:** 2016-04-28

**Authors:** Shaheen Kanthawala, Amber Vermeesch, Barbara Given, Jina Huh

**Affiliations:** ^1^ Department of Media and Information Michigan State University East Lansing, MI United States; ^2^ School of Nursing University of Portland Portland, OR United States; ^3^ College of Nursing Michigan State University East Lansing, MI United States; ^4^ Department of Medicine University of California San Diego La Jolla, CA United States

**Keywords:** health communication, online health communities, question types classification, self-management, health-related Internet behavior use, risk of misinformation, Internet, diabetes

## Abstract

**Background:**

About 6 million people search for health information on the Internet each day in the United States. Both patients and caregivers search for information about prescribed courses of treatments, unanswered questions after a visit to their providers, or diet and exercise regimens. Past literature has indicated potential challenges around quality in health information available on the Internet. However, diverse information exists on the Internet—ranging from government-initiated webpages to personal blog pages. Yet we do not fully understand the strengths and weaknesses of different types of information available on the Internet.

**Objective:**

The objective of this research was to investigate the strengths and challenges of various types of health information available online and to suggest what information sources best fit various question types.

**Methods:**

We collected questions posted to and the responses they received from an online diabetes community and classified them according to Rothwell’s classification of question types (fact, policy, or value questions). We selected 60 questions (20 each of fact, policy, and value) and the replies the questions received from the community. We then searched for responses to the same questions using a search engine and recorded the

**Results:**

Community responses answered more questions than did search results overall. Search results were most effective in answering value questions and least effective in answering policy questions. Community responses answered questions across question types at an equivalent rate, but most answered policy questions and the least answered fact questions. Value questions were most answered by community responses, but some of these answers provided by the community were incorrect. Fact question search results were the most clinically valid.

**Conclusions:**

The Internet is a prevalent source of health information for people. The information quality people encounter online can have a large impact on them. We present what kinds of questions people ask online and the advantages and disadvantages of various information sources in getting answers to those questions. This study contributes to addressing people’s online health information needs.

## Introduction

On average, about 6 million individuals in the United States search for health information on the Internet per day [1]. This number is greater than the 2.27 million physician office visits per day [1].

People look for health information on the Internet as patients or as caregivers [2]. They look for information such as a newly prescribed course of treatment, unanswered questions after visiting providers, or information about a change in diet or exercise habits [3]. Patients consult the Internet over their providers’ suggestions, or challenge their providers’ suggestions based on information these patients find on the Internet [4]. Most people who find the information they were looking for believe it is of good quality and trustworthy [5]. This could prove to be potentially problematic for situations such as when health information seekers with low health literacy skills are unable to evaluate the information they find [6]. Past research has noted the need for consumer education on Internet navigation and suggested the incorporation of decision aids into health information websites [7].

The Internet provides users access to a myriad of health-related sources, such as government and professional organization websites, medical journals, mailing lists, articles, and online support groups [5]. The Internet can put a person in touch with others with similar conditions. Especially in cases where the patient has a chronic illness, where a large part of the disease management occurs at home, social media environments provide a primary resource for people to get help from peer patients [8]. According to a Pew Research Center survey [9], 23% of patients online who have chronic illnesses have used the Internet to find others with similar health conditions. These patients found getting emotional support and quick remedies from fellow patients helpful [10]. Thus, the Internet provides a variety of resources that are advantageous for one situation over another.

In this study, we compared how 2 frequently used sources of health information on the Internet—answers from other patients (eg, replies in online health communities) and Internet search results (eg, querying search engines such as Google.com)—have unique advantages and disadvantages. We discuss implications for how online health information seekers can be further supported to receive high-quality information. Depending on the types of questions health information seekers have, search engines could provide more helpful resources than would online health communities, or vice versa. Identifying the most appropriate Internet health information resource is challenging for patients. We addressed this challenge by investigating what informative sources are most appropriate for the types of questions information seekers have.

### Background

In 2013, the Pew Research Center found that 8 out of 10 health-related inquiries started with a search engine [2]. About 77% of online health information seekers stated they used websites such as Google, Bing, or Yahoo. Of the survey respondents, 13% visited specialized websites such as WebMD, 2% visited more general websites such as Wikipedia, and 1% began their online health information seeking with a social network through websites such as Facebook [2].

In 1996, the National Library of Medicine had reported that 7 million of their annual Internet searches were health related [11]. In 2003, Google reported that 6.75 million of their daily search logs were health related [11]. In 2012, Google accounted for around two-thirds of US Internet searching, and this share is increasing [12]. These statistics show the exponential growth of Internet health information seeking.

The Internet provides a resource for patients who have similar health conditions to connect with one another. Connecting with other patients provides them with an outlet to share similar experiences and receive informational and emotional support [3,10,13,14]. A group of researchers studied what patients posted in online health communities to understand patients’ information needs from such communities [3]. Patients needed expertise coming from clinicians as well as expertise coming from fellow patients. Some patients also posted “desperate calls for help,” such as what to do when their blood sugar monitors detected extremely high blood sugar [3,15]. As such, questions about their health concerns vary greatly. One might ask factual information about a medical condition, others’ opinions about a certain recipe, or how to maneuver through a side effect.

Researchers approached taxonomy of questions in several ways based on their study context and the purpose in classifying questions [16-19]. One taxonomy of questions classified them based on the individual’s domain knowledge level. For instance, a novel situation is where the person is unfamiliar with the domain. Thus, the person lacks prior knowledge of how to approach the problem. Misindexed knowledge refers to when the individual has prior knowledge but this information has not been correctly classified under the cues for a particular schema. Incorrect or incompletely understood knowledge is when previous experience and knowledge may have been incomplete or incorrect [16].

Based on tutoring transcripts, Nielsen et al [18] developed a taxonomy of questions built on whether the question was asking for a description, explanation, comparison, or preference. They then used these question types to automatically generate questions for educational assessment [18]. Ely and colleague’s [20] evidence taxonomy helped to identify clinical questions requiring answers with evidence and whether the question was specific to an individual patient. Tutos and Mollá [19] applied this evidence taxonomy to identify clinical questions in a search engines context.

For questions asked in a social medium, Efron and Winget [17] developed question classification in the context of microblogging (eg, Twitter.com). They organized questions into 9 categories that address the purpose of the question being asked more than the type of question. For instance, Efron and Winget described some questions asked in microblogging as rhetorical in nature, where the questions invited action or coordinated action among the participants of a particular microblogging thread.

For our purpose of identifying the types of questions asked by patients and caregivers online, we needed a classification schema that could encompass the variety of patients’ and caregivers’ information-seeking needs as discussed in prior work [3]. Patients’ and caregivers’ questions posed online can be unstructured and incomplete [3]. The question taxonomies discussed so far were limited for our purpose for two reasons: the taxonomies covered only a subgroup of question types seen in patients’ and caregivers’ questions online; or they assumed that the questions were structured and well formulated.

Rothwell’s classification of questions [21], primarily designed to understand questions asked in small groups, most appropriately addressed these specialized needs of classifying patients’ and caregivers’ questions online. Rothwell argued that questions could be phrased as *fact*, *value*, or *policy* questions. A question of fact asks whether something is true and to what extent. This question can be answered with the help of objective evidence. A question of value asks for an evaluation of the desirability of an object, idea, event, or person. Such a question cannot be answered with objective evidence, since these answers are subjective views of the responder. A question of policy asks whether a specific course of action should be undertaken to solve a problem [21].

To extract patients’ questions online, we used patients’ posts from online health communities. WebMD, one of the top 1000 websites worldwide, reported in 2012 [22] that they had 111.8 million unique monthly visitors out of the estimated 117.8 million unique monthly visitors to all general health-related sites [23]. This website was one of the most popular health discussion boards for patients available online with about 19.5 million visits as of December 2012 [24]. The website consists of multiple health communities where people ask questions and get responses from the community members.

### Study Objective

Questions posed to these communities provide insights into the types of questions patients have about their health issues. Because of the diverse content of patients’ questions, what constitutes an optimal source and content to answer those patients’ questions can vary greatly. We used patients’ questions posted on the WebMD diabetes community to understand how those questions can benefit from 2 main sources of health information on the Internet: a search engine versus responses from peers in online health communities. Our research questions were (1) What health information do search engines provide versus online health communities? (2) How clinically accurate is information in search engines versus that in online health communities? (3) What is the most appropriate source of health information for different question types?

Below, we describe how we operationalized these research questions.

## Methods

### Collection of Questions

We collected patients’ questions and community responses from the WebMD online diabetes community. We chose the diabetes community over other communities because of the balanced amount of questions across various question types a diabetes context allows [3]. We wrote a script to download publicly available WebMD online diabetes community posts to a local MySQL database (version 5.6, Oracle) with a Sequel Pro interface (open source software under MIT license). Our institutional review board decided that our study did not require their regulation because the data were equivalent to public observation.

Our dataset contained 71,177 community posts between 2007 and 2014. These consisted of 9576 thread-initiating posts and 61,592 replies to those posts. The thread-initiating posts contained patients’ questions, emotional support-seeking messages, or information dissemination [3]. From our prior work, we learned that thread-initiating posts with shorter lengths included more patient questions than longer posts, which tended to be rapport building. We filtered the data down to 1555 thread-initiating posts with fewer than 200 characters, that is, short posts. Next, to examine the most recent questions posted by patients, we organized the posts by posting date. We coded down the list from the most recent to older posts coding for (1) whether the post was a question and, if so, (2) which type of question it was based on our codebook.

We iteratively modified Rothwell’s classification of questions to develop the following codebook:

- Questions of fact: These questions ask whether something is true and to what extent, requesting objective, factual information (eg, “What are the normal ranges for blood sugar?”; “What could it mean if you have a sweet taste in your mouth?”).
- Questions of policy: These questions ask whether a specific course of action should be undertaken to solve a problem (eg, “I just got diagnosed with type 2 diabetes. What should I do next?”; “After overnight fasting I experienced a sugar spike. What should I do to bring it back down?”).
- Questions of value: These questions ask for an evaluation of an idea, object, or event of a person (eg, “Has anyone experienced tingly fingers as a side effect to diabetes?”; “Can someone describe their experience with foot surgery and healing associated with this kind of surgery?”).

We then selected the most recent 100 thread-initiating posts. One coder (henceforth referred to as the nonclinical coder) started to code the 100 posts for whether the post was a question, starting with the most recent. If it was a question, this coder coded the question type. The coder continued the process until we had 20 questions under each question type. As a result, we had 60 patient questions in total to investigate. Half of the questions from the total of 60 questions were randomly selected and given to another coder to measure interrater reliability between the 2 coders.

The average interrater reliability between the coders was 0.79. We then convened to resolve the disagreement. We dropped the disagreed item and recoded until we found another fact question to have an equal number of questions for each question type, which resulted in 20 fact questions, 20 value questions, and 20 policy questions.

### Collection of Search Engine Results

To determine what information a search engine would retrieve, we queried each of the resulting 60 questions on Google.com. We chose Google.com because it is the most used search engine as of 2015, according to websites that measure search engine traffic (ie, alexa.com and amazon.com) based on combined measure of page views and unique site users [25]. We used the full sentence of each question as the query to mimic how an online health information seeker asks questions through a search engine [26]. Additionally, we chose to keep the questions as they were posed to keep the sentiment of the question intact, that is, whether it was a fact, a value, or a policy question.

User searches comprise both keyword-based searches and sentence-based searches. Older search engines, such as AltaVista or AOL, were based on the model of keyword-based searches. With advancing Internet use, search engines recognized supporting sentence-based searches, especially in the context of users asking for answers to their questions. Consequently, Microsoft developed a patent on parsing searches of Frequently Asked Questions pages [27]. Hence, while keyword searches might be more prevalent for general search engine use, for the specific user needs context we are addressing—asking questions on the Internet—a sentence-based search model is more appropriate. Thus, we used the questions posted in online communities to address the types of search queries that users would post in the context of getting answers.

Research has shown that people explored only the first few results through a perfunctory examination of the search results [28]. One study used eye tracking to determine how the ranking of a link in search results affected the amount of attention it received. Their results indicated that people spent almost equal time looking at the first and second links—the viewing attention span drastically dropped from the third link onward. However, substantially fewer participants clicked the second link than the first [29]. To mimic people’s practices around reading Internet search engine results, we limited our data collection to the first 3 search results for each question. We excluded search results that linked back to the WebMD online health community, because this information source would be a duplicate of our other set of data on the community responses. Searching for information through a search engine directs users to WebMD and, therefore, direct question searching on the Internet could lead individuals to information and discussions in online health communities, such as this one.

Because each question had 3 online search results, each category resulted in 60 search results. However, some searches yielded fewer than 3 results, while others resulted only in the original WebMD question and its responses. This explains the lower than 60 search results (57 results) in the fact-type questions below.

### Collection of Community Responses

For each question, we returned to its original post in the WebMD community and collected the responses to these posts. The number of responses to these posts varied from 0 to 30 each. We collected all of these responses, including the responses to other responses. We did not restrict the total number of community responses per question for the analysis based on the finding that online health community users attempt to read all replies to the question unless the user deems the replies to be unrelated to the topic.

### Analysis of Search Results and Community Responses

For each search result and community response, the nonclinical coder answered the following questions: (1) How complete was the answer? (2) What kind of information did it or did it not provide? Then, we organized these observations as advantages and disadvantages for each search result and community response. The second nonclinical coder followed the same analysis process for one-quarter of the search results and community answers. The 2 coders examined each other’s analyses and discussed disagreements. The resulting discussion informed the first nonclinical coder’s analysis for the rest of the search results and community answers. The third coder—a family nurse practitioner—answered the following questions for all community answers and search results: (1) Did the information answer the questions? (2) How clinically relevant is the information? and (3) How clinically valid is the information? The third coder was also given a space for adding open-ended, qualitative comments. This clinical coder will be referred to as clinician A from here onward.

We defined *clinically relevant* as clinical information pertinent to the question asked. Even if the information did not answer the question, if it was relevant to the question, we considered the information as clinically relevant. We defined *clinically valid* as the accuracy of the information provided to the posed question, based on the knowledge of a clinician. Even if the information did not answer the question, the information was clinically valid if it was accurate. Through discussion with clinician A, we concluded that the factors influencing this coder’s decision on whether a piece of information was clinically valid included the accuracy of the information on the website, the context from which the patient was posting the question, the safety (ie, level of potential harm to the patient) of the information presented, the health literacy level of the website, and whether the website was advertising a product. The website’s mode of delivery was also considered, along with whether websites required additional clicks to follow links or download videos or PDFs.

We also had a fourth coder (henceforth referred to as clinician B)—a nursing faculty member with credentials as a registered nurse and a Fellow of the American Academy of Nursing. We provided this fourth coder a random sample of 15 questions from the total 60 questions to assess agreement between the clinicians. Both clinicians followed the same method of coding search results and community findings. Once the coding was completed, we discussed disagreements and common themes among the results.

## Results

In this section, we walk through the content of the collected questions for each question type. We then describe our coding results. Multimedia Appendix 1 presents sample questions for each question type, alongside search results and community responses.

### Question Content Under Each Question Type

In this section, we describe what kinds of questions our dataset included under each question type. *Fact questions* asked about factual information regarding diabetes medications and their effects (7/20 questions), fluctuating blood sugar level issues (7/20), diet and exercise and their effects on diabetes (4/20), blood pressure levels (1/20), and diabetes types (eg, brittle diabetes) (1/20). *Value questions* asked about people’s experiences with medication or medicinal devices (12/20), food products or diet supplements for diabetes (3/20), diabetes-related symptoms (3/20), and other illnesses such as a stomach virus or a suspicious mammogram (2/20). *Policy questions* asked what course of action should be taken regarding medication and the side effects caused by diabetes and its medication (7/20), blood sugar levels (6/20), diabetes treatments (5/20), weight loss (1/20), and diet (1/20).

### Analysis of Search Results and Community Responses for the 3 Question Types

#### Fact Questions: Most Clinically Valid Search Results

The search results for fact questions provided a variety of information sources, including video, question-and-answer websites containing health care providers’ answers to patients’ questions, overviews of factual information about the requested topic, examples for information seekers to follow, and weblinks to other potential resources, including well-reputed sources such as the American Diabetes Association website. Some even led to online tests to help users determine an answer to their question (such as a prediabetes test). Multimedia Appendix 1 provides a complete example of a question along with all of its search results.

Among these search results, clinician A identified 19/57 results to be correctly answering the questions. The rest were coded as incorrectly answering the questions due to the following reasons: the information was tangentially relevant, it did not directly address the question, the answer was incorrectly phrased, it appeared on an unreliable tabloid or advertisement webpage, or the information was outdated or old. The clinician also commented that accessibility to information was a potential challenge in information provided by the search results, such as in the case of large videos that take a long time to download and highly technical resources that add complications to understanding the material.

Clinician A also found 28/57 search results to be clinically relevant and 37/57 to be clinically valid. For instance, the question “Can Insulin alter the efficacy of Coumadin therapy?” led to a search result about what Coumadin is. This page provided an overview of the generic form of the drug (warfarin) and its brand name drugs, including Coumadin. This page was coded by clinician A as clinically relevant but not clinically valid. This was because the information on the page was correct, according to the clinician, but did not answer the question.

Our analysis showed that these search results provided a varying quality of information in terms of how much the information answered the questions: some partially addressed the question, and others provided a complete and comprehensive answer, while some gave an overview about the question’s topic but did not fully address it. For instance, a poster’s question was “Is Byetta a non insulin medication and can it be taken with Metformin? [sic].” To this question, the top search result was a website containing information about oral and noninsulin injectable medications for diabetes. This website contained information on Byetta being a noninsulin medication but it lacked information on whether it could be consumed with metformin, which was part of what the question was asking.

Some search results did not answer the questions at all. These results included information irrelevant to the questions with large amounts of text on the page, which would be overwhelming for lay users. The search results coded as not answering questions by both the nonclinical coder and clinician A presented extraneous information such as a class study guide, commercials for products, or a response to the question but not in a diabetes context. Some search results also did not include sources or citations from which the information was derived, thereby making the validity of the information questionable.

Community responses for fact questions contained personal experiences, anecdotes, and assessment of information provided by other responders. The community responses also included reassurances and compliments to the poster, psychosocially supporting them. For instance, to the question “I am new to diabetes. I have noticed that my blood sug[a]r goes to 180–200 when I exercise. The more strenuous the exercise the higher the blood sug[a]r. Does anyone know why this happens?”, a poster responded: “Exercise is a form of stress on the body. Whenever you have stress, your liver secretes sugar. Even though it goes up during exercise, you may notice it dropping low after you are done. This does happen to me too, and I make sure to drink plenty of water while I work out.” This conversation describes how a community response answers a poster’s question while also providing personal experience with the problem and a way to resolve it based on the responder’s own method. This was the most critical difference between the search results and community responses. Another prevalent answer to posters’ questions was responders asking the posters to discuss their questions with their health care providers in case it was something that needed medical intervention. The responders also assessed the accuracy of other people’s responses. Responders also denied answering questions due to the liability of the community.

Clinician A determined that 35/66 community responses answered fact questions. Similar to fact question search results, those community responses coded as not answering questions included general encouragement for the poster only (for instance, “it is great that you are being proactive about your health now”), responses to conversations with other individuals involved in the post, or just responses that were clinically incorrect. Additionally, 37/66 of the community responses were both clinically relevant and clinically valid.

Fact question community responses not only advised a poster to visit a health professional, but also provided suggestions about what should be discussed during this visit, referred posters to external resources, gave compliments and reassurances about the difficult time the poster was going through, and even alerted a poster to incorrect or dangerous information provided by other responders.

Overall, community responses presented more information related to answering the question content than did the search results.

Finally, we calculated the interrater reliability of clinicians A and B using Cohen kappa score. The overall kappa for fact questions was .46, indicating a weak level of agreement between the 2 clinicians [30]. A more detailed discussion explaining this lower interrater reliability is at the end of the Results section.

#### Policy Questions: Most Answered Through Community Responses, Least Answered Through Search Results

Search results of policy questions included diabetes management pages, blogs, stepwise instructions on how to solve a problem, and patient forums. However, not all answers were available within an article reached through a search result—often, the information was present (fully or tangentially) in the discussion or comments section of the page. Because these were how-to questions, the search results also led to different social media platforms such as Facebook, where other similar questions fully or partially answered the poster’s question. For instance, the question “hi i just checked my blood sugar and its 490 how can i get it down my vision is blurry? [sic]” leads to a Facebook type 1 diabetes page post discussing eyesight fluctuation for a 25-year-old with newly diagnosed type 1 diabetes. This post is not an exact answer to the poster’s query, but it partially explores the poster’s questions. Furthermore, the comments the Facebook post received could help the poster get his or her answer. Some results did not answer the questions asked.

Our coding results indicated that a very low number of policy questions were answered through search results. Among the search results of policy questions, clinician A identified that 8/60 results correctly answered the questions. This coder also found that 8/60 search results were clinically relevant but 24/60 were clinically valid.

A search engine does not directly answer “what or how should I do [something]?” questions. For instance, a person asking about how to lower blood sugar first thing in the morning, due to high early-morning numbers, is led to a page describing the “dawn phenomenon”—a condition many diabetics experience wherein their morning sugar levels are higher than usual [31]. This helps patients with the information, but would not necessarily answer their question of what to do to remedy it.

Other search results included discussion pages related to the question posed, a stepwise to-do response to the question (such as ways to lose baby weight), external resources, advice on what the poster can do next (for instance, the next steps of having diabetes diagnosed), and access to social media pages displaying similar situations to the poster’s.

Community responses to policy questions provided personal experiences with similar problems and gave posters insight on how to deal with their problems. These responses also prompted posters to think about other questions related to the situation, advised them to visit their health care provider, provided additional resources, reassured posters that they were not alone, and made them aware of potential dangers. For instance, to the question “is anyone on that can tell me how to lower my blood sugar. What can I eat right now to lower it. I had to many carb’s and it’s 202 usually it’s 90 to 101 [sic]”, a response provided stated “Hi, The only way to get your BG lowered from a spike is to exercise and drink a lot of water. There are no foods to bring your glucose level down. At least this is my meager experience and if someone else has a better idea I hope they share with you. Good luck and watch those carbs [sic].”

Clinician A observed that 30/49 community responses answered the questions posed, 30/49 responses were clinically relevant, and 31/49 responses were clinically valid. This imbalance between clinical relevance and clinical validity occurred because 1 poster posed a question about having a blood sugar level of 490 and asked how to bring it down so as to get rid of blurry vision. A responder suggested this person should wash his or her hands and try again and, if the sugar reading stayed as high, should call 911 or head to the emergency room immediately. This post was marked clinically valid because all the information it provided was clinically accurate; however, it was not marked as clinically relevant, because the information was not relevant to the actual question asked and did not help answer it (ie, how to get rid of the blurry vision).

Other community responses to policy questions stressed the dangers of a situation a poster may be in; provided potential solutions to help solve a problem, including suggesting home remedies and advising the poster to visit a health professional; offered personal stories, anecdotes, and opinions; and helped detect emergencies from the situation presented by the poster.

Finally, we calculated the interrater reliability of clinicians A and B using Cohen kappa score. The overall kappa for policy questions was .52, indicating weak agreement between the clinicians [30].

#### Value Questions: Community Responses With Mixed Quality Assessment by Clinicians

Search results for value questions included personal experiences and, therefore, a lot of the search results only partially answered the question or provided an overview of the subject. Search results included products and their reviews, patients’ stories through blog posts, and discussion forums. One result helped with alleviating nervousness, while another showed how others also had similar symptoms. A portion of the questions asked about people’s experiences with medicine or health issues (as elaborated in detail above); therefore, the search results led to online stores and reviews for these products, such as Amazon, where other customers provided reviews for the health information seeker to review. Lastly, some questions were answered, but not necessarily in a diabetes context. For instance, a poster’s question was “I had my first [mammogram] last week and it came back suspicious. Had to go for more [picture] and an ultrasound and now for a biopsy. I am way past scared to death. Can anyone help me [sic]?” the first search result to this question led to a webpage discussion about mammograms in patients. This information provided encouragement to the poster as requested.

Among the search results of value questions, clinician A identified that 23/60 results correctly answered the questions. This clinician also found that 23/60 search results were clinically relevant and 29/60 were clinically valid. These results indicate that, while some questions were answered, still others were clinically relevant and clinically valid, but did not answer the question.

Other search results for value questions included a comprehensive overview of the subject in question; discussed side effects from credible sources; had comment sections on some webpages discussing the subject; helped differentiate between myths and facts related to the question; led to question-and-answer pages that help answer the question posed; resulted in product reviews for questions about specific products, thereby informing the poster about the quality and effectiveness of the product; included personal experience stories and encouragement, which reduced posters’ nervousness; and included some results that were also backed up with statistical evidence.

Community responses, on the other hand, included details about things the question poser should be cautious about, provided side effects of medication, tips, and suggestions, conducted online searches, and found answers for the poster.

People are enthusiastic about providing their opinion. The downside of this is that there is no way to verify the answers provided. For instance, a poster posed the question “Have any of you had the Bayer Contour meter just readout “HIGH”? No numeric reading just “HIGH”. I suspect that is a very bad sign [sic].” A response to this was, “Most meters read up to about 500 or 600. Anything higher than that and it simply greets you and says “HI”. Did you test again after washing your hands? If you did call your doc and see someone immediately. This is not good [sic].”

For community responses, clinician A noted that 63/104 responses provided answered the questions posed. Additionally, 51/104 were clinically relevant and 47/104 were clinically valid.

The types of community responses received by a poster for value questions helped them be wary about new trends; informed them about medicinal side effects and provided insights about how this information was obtained; alerted people to any potential dangers; provided personal experiences, opinions and anecdotes, and tips and solutions to help resolve issues; advised the questioner to visit a health professional; corrected or clarified misinformation; redirected the poster to more resources and information; and reassured and encouraged the poster about his or her current situation.

Finally, we calculated the interrater reliability of clinicians A and B using Cohen kappa score. The overall kappa for fact questions was .46, indicating a weak agreement between the 2 clinicians [30].

### Clinician Interrater Reliability

As can be noted, the Cohen kappa score between both clinicians was not very high, signifying weak interrater reliability. Some of the difference in coding between them can be explained through the following reasons. First, we found many of the search results on webpages containing large amounts of text, thereby making the process of locating the response on the page a difficult one. This sometimes led to 1 clinician coding the search result as answering the question posted, whereas the other did not. This observation depicts how different people, including clinicians, interpret different kinds of search results in terms of whether they answer the question asked. If both coders coded a question differently, the subsequent questions about clinical relevancy and clinical validity also tended to follow different paths. Sometimes, in a situation like this, the search result was coded as answering the question, but the validity or relevance of the answer was queried, therefore leading to different codes between the 2 clinicians. This variability in the results between the 2 clinicians speaks to the complexity of the problem, that is, the difficulty of defining and assessing the quality of information on the Internet.

Finally, the 2 coders interpreted the accuracy and relevance of some responses differently, therefore leading to lower kappa scores. This result points to the importance of the way information is shared and interpreted on the Internet and how better guidance and direction for gathering information is necessary. This study contributes to understanding the various factors clinicians consider and how these factors lead to their evaluations.

Table 1 summarizes the advantages and disadvantages of both sources. These are the characteristics of the overall findings of each category—every point does not apply to every finding. This is followed by an overview of clinician A’s findings in Table 2.

**Table 1:** Summary of advantages and disadvantages of search results versus community responses to questions posted to an online diabetes community.

Type of question		Advantages	Disadvantages
**Search results**			
	**Fact**		
		Provides some answers to the questions.	Does not always provide an answer to questions posed.
		Provides an overview of the subject.	If question is answered, it could be only a partial response.
		Multiple websites provide a wide range of information.	Can provide irrelevant responses.
		External links can route health information seekers to various resources.	Can answer out of the context it is posed in (ie, out of a diabetes context).
		Responses from reputed websites, such as the American Diabetes Association, can be assumed to be accurate.	Accuracy of responses is not always known.
		Provides a test to help posters determine their answer (eg, a prediabetes test).	Websites can contain large amount of content, thereby preventing easy location of response.
			Some results are commercials for products, leading to biased information.
			Does not answer unusual questions.
	**Policy**		
		Gives access to the discussion and conversation pages related to the question posed.	Some results do not answer the question directly, leaving the question poser to make extrapolations.
		Provides step-by-step responses to the questions posed (eg, ways to lose baby weight).	Some results do not provide required answers, ie, are irrelevant.
		Provides external resources to relevant information.	Some results answer questions partially or tangentially.
		Provides the next steps for poster (eg, next steps of having a diabetes diagnosis).	Websites can contain a large amount of content, thereby preventing easy location of response.
		Provides access to social media results, such as Facebook, showcasing similar cases.	Some results are commercials, leading to biased information.
	**Value**		
		Some responses answer questions precisely.	Some results do not provide required answers, ie, are irrelevant.
		Some results provide a good overview of the question topic.	Some results answer questions partially or tangentially.
		Some responses discuss side effects from credible sources.	Website can be very large and contain a lot of content, thereby preventing easy location of response.
		The comments sections of webpages help discuss the subject.	Some results are commercials, leading to biased information.
		Answers differentiate between myths and facts of the subject.	
		Question-and-answer pages help answer poster’s question.	
		Some pages lead to product reviews that help answer the question.	
		User experiences and encouragement on different result pages help alleviate poster’s nervousness.	
		Some results back up claims through statistical evidence.	
**Community responses**			
	**Fact**		
		Provides personal experiences, opinions, and anecdotes.	Some questions do not get responses.
		Advises poster to visit a health professional.	Cannot check accuracy of responses.
		Provides examples and external resources.	Question may be deferred to a health professional, thereby delaying response.
		Provides compliments and reassurances for the difficult time the poster is going through.	Some questions are answered only partially.
		Alerts poster of potential dangers (including those from other people’s responses).	Cannot answer due to liability of the forum.
		Redirects to a person or resource with more information.	Does not always provide a complete or relevant response.
		Provides alternative options, external resources, and potential talking points to discuss with one’s health care professional.	Some responses are irrelevant or potentially dangerous.
	**Policy**		
		Responses stress the dangers of the situation.	Some questions do not get responses.
		Provides tips or solutions to resolve issue.	No way to check accuracy of responses.
		Provides personal experiences, opinions, and anecdotes.	Some questions are answered only partially.
		Advises poster to visit a health care professional.	Some responses are not in line with other responders.
		Provides home remedies.	
		Detects emergency cases.	
	**Value**		
		Some results help posters be wary of latest trends.	Some questions do not get responses or are irrelevant.
		Some results provide effects and side effects along with insights about how this information was found.	
		Alerts poster to potential dangers.	No way to check accuracy of responses.
		Provides personal experiences, opinions, and anecdotes.	Some questions are answered only partially.
		Provides tips or solutions to resolve issue.	
		Advises poster to visit a health care professional.	
		Responses help clear misinformation for poster.	
		Redirects to a person or resource with more information.	
		Provides reassurances and encouragement to poster.	

**Table 2:** Evaluation of all community answers and search results by Clinician A.

Type of question		Answers the question		Is clinically relevant		Is clinically valid	
		No.	%	No.	%	No.	%
**Fact**							
	Search results (n=57)	19	33	28	49	37	65
	Community responses (n=66)	35	53	37	56	37	56
**Policy**							
	Search results (n=60)	8	13	8	13	24	40
	Community responses (n=49)	30	61	30	61	31	63
**Value**							
	Search results (n=60)	23	38	23	38	29	48
	Community responses (n=104)	63	61	51	49	47	45

Table 2 breaks down the questions according to clinician A’s analyses. This table lists the proportion of positive responses to the selected questions in each category, that is, whether the results or responses answered the questions, were clinically relevant, and were clinically valid. For example, 19 of the 57 observed search results for fact-type questions answered the question posed. The remaining results either did not answer the question posed or contained unknown or not applicable information, such as webpages that would not open or community posts with no answers.

For example, the question “recently lost a kidney/ureter to cancer. sugar levels are moderately changed and i need to know the actual effects i can expect as my body adjusts to life with only one kidney [sic].” leads to a human anatomy textbook online, which clinician A deemed to have an “unknown” value.

The community responses, on the other hand, varied in number, since we included every answer to a question posed. The total numbers indicated above are the number of responses each category of community responses received. However, there were questions with no responses at all, such as the question “Help. I just gave myself my pre dinner shot in my leg. It formed a huge bump on my leg. Will the insulin still get in me or do I need to take my shot again???[sic]” had no responses at all and therefore clinician A marked it as “not applicable.”

## Discussion

### Principle Findings

Our analyses showed that community responses answered more questions than did search results across the board. These community responses were more clinically relevant than their search result counterparts. However, clinical validity varied, with search results being more clinically valid than community responses for fact and value questions, but community responses being more clinically valid than search results for policy questions. These observations show that answers from both sources contain clinically accurate information, which does not necessarily answer questions.

Search results for fact questions showed that results were often clinically valid but not clinically relevant. Fact question search results had the highest validity among all question types. Even if search results were clinically relevant, only a small portion of those results completely addressed the questions in the query. Questions left unanswered can be attributed to imprecise question phrasing. Wording questions so that their meaning is clear and concise will lead to more relevant search results than will questions with meandering and unclear content. We need mechanisms to help patients formulate queries and questions online.

We learned from the search results of policy questions that nearly half of the search results to policy questions contained clinically valid information. However, most of this information was irrelevant to answering the questions and the information was incomplete. A reason for this finding could be the way policy questions are framed. These questions require information about how or what needs to be done in a particular situation. Factoring in the multiple variables in an individual’s question would result in varied search results, none of which answer the question as required.

Analysis of value question search results showed that search results often did not answer the question posed, nor was the information clinically relevant. However, a greater number of results were clinically valid, showing that accurate information does not always lead to answers. This observation is in line with fact and policy question search results, where a larger number of results were clinically valid but did not answer the question posed.

We observed a similar pattern in the community responses to fact questions, where a greater number of questions were clinically valid and clinically relevant, but fewer questions were answered.

For community responses to policy questions, clinician A observed that a much higher number of questions were answered as compared with policy question search results. This shows how the specific nature of policy questions makes them a better fit for an audience who are familiar with the issues of the community, thereby providing posters with the information required.

Community responses to value questions were opposite to fact and policy community responses. Value questions were the only question type to which the community provided a greater number of answers, but a smaller fraction of the information provided was accurate. In comparison, fact and policy questions tended to get superfluous but clinically valid responses, leading to more unanswered questions. This finding is important because it portrays the risks of unchecked information being exchanged in online health communities. Value questions ask others for their personal experiences and evaluations, which is a warning to posters about the unmoderated nature of the information.

The combined analyses of the coders and the clinicians indicated that policy and value questions get more community responses than do fact questions. This observation could be because these 2 categories ask responders to provide their personal experiences with a situation, and knowing an answer to a question or not (as with fact questions) is not a criterion.

Community responses also make question posters feel better about their situation and remind them that they are not alone through reassurances and compliments. The responses warn question posters about potentially dangerous actions or incorrect advice they get from other responders.

We also observed that community responses consistently got a higher percentage of questions answered. as opposed to a search results. Therefore, while past research indicates that people go online and search for health-related information, these individuals are more likely to get their questions answered through online social support groups of people in similar situations to their own.

### Recommendations

Based on the advantages and disadvantages of the search results and community responses (Table1), there are potential recommendations for stakeholders involved.

Patients must be vigilant about the information they find through search results by keeping track of the sources of websites and the validity of the information provided. Formation of the question asked in search results also plays a role in the kinds of responses it gets; therefore, posing a clear question while searching for results on the Internet is important. Community responses are provided by similar others, that is, other individuals facing health situations similar to the question poster’s. While this indicates a familiarity with a question posed or a situation described, it is important to verify medically related information or steps to be taken with one’s health care provider so as to prevent negative health consequences.

Guiding patients toward accurate information obtained through search results helps providers by not having to correct misconceptions patients build through information they gather via search results. It would be helpful to patients for providers to guide them in searching for information online and in determining whether information is trustworthy.

Online health communities play a critical role in providing social support to people going through similar health issues. Self-management of health conditions plays an important role in management of chronic illnesses [32], such as diabetes. Introducing patients to such a resource and encouraging them to use it is helpful, for both the patient and the provider. Patients can build a network of resources additional to their providers—a support system that is available to them, preventing them from feeling isolated and being frightened of their situation. Providers can get additional time that can be allocated to patients in greater need or to themselves.

Researchers should compare different online communities to analyze information sharing for a variety of health conditions. Such health information can help technology developers create more efficient health communities, with more opportunities and resources for patients participating in them. One important finding was the sharing of incorrect or dangerous information by other participants in an online community. Developers could create a way to tag dangerous posts, based on feedback provided by other users, which would require moderators of the community to evaluate such posts and rectify the information provided as needed. Classification of questions based on our codebook may help researchers and developers in the future to tag questions needing professional evaluation. Answers to policy questions provide direction to individuals in need, and value questions give personal evaluation, both of which we have observed to have incorrect information. In future studies, researchers should also focus on the best way to formulate questions to gain the most accurate information through search results. Additionally, it is important to develop a way to help patients analyze whether the information they gather through Internet search results is accurate. Patients look for health information online in large numbers. Therefore, ensuring the accuracy of this information is crucial.

Informaticists should analyze the way information is shared in online health communities, especially through the relations between participants within these communities. Participants roles in these communities provide insights into how relationships form and how these relationships lead to an exchange of information. These insights include the kinds of information they share and receive, and whether it is clinical or social in nature. Additionally, informaticists should look into the questions posted, both those searched for on the Internet and those posted in online communities. Such an analysis would prove valuable to determine which questions would be ideally suited to be answer through a search engine versus those ideally posted in an online community (ie, which would provide the most accurate and complete information to a specific question).

Finally, the trouble faced by both of our clinicians in interpreting similar information differently is a commentary on how difficult it is to find and assess health information online. This observation is important so future websites can address this wide-ranging quality issue.

### Limitations

As mentioned above, because the questions posted in the search engine came from a diabetes health community, not all of the questions mention their diabetes context. Community members assume this diabetes context. For example, an evaluation of a particular diet would not ask members to keep in mind that the diet was for a person with diabetes because it was posed in a diabetes community. Because we decided to search unaltered questions in the search engine, the answers we got could have been less efficient (answers not in a diabetes context) than if we had modified the questions to include this parameter. Future studies could make the context explicit to study the differences in answers it would produce.

Searching complete questions could misguide a search engine. Future research can focus on alternatives such as carrying out keyword searches with pertinent information from the question as opposed to using the question as an exact-phrase search.

Additionally, more than 3 search results can be included in the search result answers. While most people do not venture beyond the first page of search engine results, and even there they focus on simply the first few search results [28,29], adding additional search links will give a comprehensive insight into the kind of responses available.

Finally, Google’s page ranking method ranks high-quality websites higher than other websites with a lower level of authority in the related topic (eg, the total number of incoming links from government institutions such as the US Centers for Disease Control and Prevention). As a result, our findings on the high accuracy of online health information in the first 3 search results are biased toward what Google already ranked as having high authority in the topic. However, most searchers check the first 2 search results, thereby correcting this imbalance [29].

### Conclusion

We evaluated the responses people get to health information they seek online from 2 different avenues: search engine results and online community responses. Our findings indicate how question types matter for determining information quality and sources. Health care practitioners, informatics researchers, and policy makers should consider the strengths and weaknesses of each information source based on the types of questions information seekers have. Our study contributes to improving online health information quality, making self-management of health more efficient and lowering costs for medical professionals and patients.

## Appendix

Multimedia Appendix 1 Examples of questions of each category and their community responses.
