# Supplementary material for: Answers to Health Questions: Internet Search Results Versus Online Health Community Responses
Source: J Med Internet Res. 2016 Apr 28;18(4):e95. doi: 10.2196/jmir.5369 (PMC4865652; doi:10.2196/jmir.5369)
Supplement: Multimedia Appendix 1 [file jmir_v18i4e95_app1.pdf]

Appendix A: Examples of questions of each category and their community responses

Example questions	Example online search results	Example community responses	
**Fact Questions**			
Can Insulin alter the efficacy of Coumadin therapy?	http://www.everydayhealth.com/drugs/coumadin	John	Hello. I did a Yahoo search of the phrase "insulin and coumadin" and got several hits. Here is a link to one article that states that there is no interaction between the two drugs: http://www.drugs.com […]. I have been on coumadin for 18 years. You really need to discuss this type of question with your doctor and/or pharmacist. Many drugs and foods do interact with coumadin and alter its efficacy.
	http://intl.pharmrev.org/content/63/2/437.full	Dan	One of the most important source of insulin is the root of the dandelion
	http://www.todaysgeriatricmedicine.com/archive/101308pe.shtml	Sara	Dan, you're not actually suggesting that dandelion root will treat type 1 are you? It would be dangerous to suggest that.
		Dan	I also find your post dangerous and have reported it as ad/spam.
**Policy Questions**			
Hi, I just checked my blood sugar and its 490 how can I get it down? My vision is blurry?	https://www.facebook.com/T1Diabetes/posts/553426361407640	Jack	If your blood glucose is 490 and you have washed your hands and checked again with similar results you should probably be calling 911 or going to the emergency room. This is not something to be messing around with. You could be headed for big trouble and just don't know it yet.
	http://www.diabetesforums.com/forum/topic/49688-how-high-will-the-glucometer-go/	James	The first poster is right. Best to test it again. Make sure your strips are not expired, too. If it truly is that high, you need a visit to the ER. That number is too high where you can pass out anywhere at any time. There is no way to get your blood sugar down within normal range unless you have insulin. If you drink lots of water, it will come down somewhat. You still will need some professional help to get it within normal range.
	http://jdrf.org/life-with-t1d/type-1-diab		

	etes-information/control-and-management/high-blood-sugar/		
**Value Questions**			
Has anyone tried Alpha Liopic Acid? Heard it is good for diabetes and other ailments. Also some doctors recommend it.	http://articles.mercola.com/sites/articles/archive/2009/05/16/this-antioxidant-can-smash-insulin-resistance-and-autoimmune-disease.aspx	Jen	I have used it to help reverse neuropathy. I know some folks take it for other reasons, but I haven't seen any good evidence (please provide some for me folks if you have some references) that suggests it helps with control or other prevention. That said, I'm not a big believer in taking a lot of stuff. I think we get what we need from natural sources in our food.
	http://www.drperlmutter.com/lipoic-acid-powerfully-effective-treating-diabetic-neuropathy/	Alex	This LEF article covers a lot of bases regarding the functions of alpha lipoic acid in the body and includes 103 references to satisfy any skeptic. If you would like additional research abstracts, this page provides seventy of them; I didn't bother checking for duplication but it gets the point across that alpha lipoic acid is an essential antioxidant in the body. Alpha lipoic acid also "recycles" or "regenerates" other antioxidants in the body (e.g. vitamins E and C)
	http://community.diabetes.org/t5/Adults-Living-with-Type-2/Anyone-here-take-Alpha-Lipoic-Acid/td-p/451001	Bill	Thanks for this info and your recent post on diabetes and Alzheimer's. I also read Life Extension information regularly. I also just called my doctor to reinstate my low dose metformin prescription for starters to see if it will help regulate my extreme glucose "awareness". Even with a low carb diet (no more than 15-20 grams of carbs at any meal and 10 at snacks) and regular exercise and weighing 135 at 5'5"
		Jen	Been out of town visiting my sisters in SoCal. I appreciate your comments and am heartened to know that someone else reads Life Extension information (I thought I was a lone wolf in these forums). Life Extension does offer very good information on diabetes but newbies often misinterpret the info as a prelude to marketing their supplements. However, the info that LE provides is

			an end point in and of themselves. There is no need to purchase anything. LE recently completed extensive work on the 5th edition of their reference work, Disease Prevention & Treatment. […]
